# Dyslipidemia and reference values for fasting plasma lipid concentrations in Danish/North-European White children and adolescents

**DOI:** 10.1186/s12887-017-0868-y

**Published:** 2017-04-28

**Authors:** Tenna Ruest Haarmark Nielsen, Ulrik Lausten-Thomsen, Cilius Esmann Fonvig, Christine Bøjsøe, Lise Pedersen, Palle Skov Bratholm, Torben Hansen, Oluf Pedersen, Jens-Christian Holm

**Affiliations:** 1 The Children’s Obesity Clinic, Department of Pediatrics, Copenhagen University Hospital Holbæk, Smedelundsgade 60, DK 4300 Holbæk, Denmark; 2 Novo Nordisk Foundation Center for Basic Metabolic Research, Section of Metabolic Genetics, University of Copenhagen, DK 2100 Copenhagen, Denmark; 3 Department of Clinical Medicine, University of Copenhagen, Copenhagen, Denmark; 4 Hans Christian Andersen Children’s Hospital, Odense University Hospital, Odense, Denmark; 5 Department of Clinical Biochemistry, Copenhagen University Hospital Holbæk, DK 4300 Holbæk, Denmark

**Keywords:** Adolescent, Child, Dyslipidemias, Lipids, Obesity, Reference values

## Abstract

**Background:**

Dyslipidemia is reported in 27 − 43% of children and adolescents with overweight/obesity and tracks into adulthood, increasing the risk of cardiovascular morbidity. Cut-off values for fasting plasma lipid concentrations are typically set at fixed levels throughout childhood. The objective of this cross-sectional study was to generate fasting plasma lipid references for a Danish/North-European White population-based cohort of children and adolescents, and investigate the prevalence of dyslipidemia in this cohort as well as in a cohort with overweight/obesity.

**Methods:**

A population-based cohort of 2141 (1275 girls) children and adolescents aged 6 − 19 (median 11.5) years was recruited from 11 municipalities in Denmark. Additionally, a cohort of children and adolescents of 1421 (774 girls) with overweight/obesity aged 6 − 19 years (median 11.8) was recruited for the study. Height, weight, and fasting plasma lipid concentrations were measured on all participants. Smoothed reference curves and percentiles were generated using the Generalized Additive Models for Location Scale and Shape package in the statistical software R.

**Results:**

In the population-based cohort, plasma concentrations of total cholesterol (TC) (*P* < 0.05), low-density lipoprotein cholesterol (LDL) (*P* < 0.005), and high-density lipoprotein cholesterol (HDL) (*P* < 0.005) were higher in the youngest compared to the oldest tertile. Fasting plasma levels of triglycerides (TG) (*P* < 0.005) increased with age in both sexes. In boys, non-HDL was lower in the oldest compared to the youngest tertile (*P* < 0.0005).

Concentrations of TC, LDL, non-HDL, and TG were higher (*P* < 0.05), and HDL lower (*P* < 0.05) in the cohort with overweight/obesity in both sexes and for all ages except for TC in the youngest girls. The overall prevalence of dyslipidemia was 6.4% in the population-based cohort and 28.0% in the cohort with overweight/obesity. The odds ratio for exhibiting dyslipidemia in the cohort with overweight/obesity compared with the population-based cohort was 6.2 (95% CI: 4.9 − 8.1, *P* < 2*10^−16^).

**Conclusion:**

Fasting plasma lipid concentrations change during childhood and adolescence and differ with sex and age. Children and adolescents with obesity have increased concentrations of circulating lipids and exhibit an increased prevalence of dyslipidemia.

**Trial registration:**

The study is part of The Danish Childhood Obesity Biobank; ClinicalTrials.gov ID-no.: NCT00928473 retrospectively registered on June 25th 2009.

**Electronic supplementary material:**

The online version of this article (doi:10.1186/s12887-017-0868-y) contains supplementary material, which is available to authorized users.

## Background

Cardiovascular disease (CVD) is still the major cause of mortality in the world [1]. Elevated concentrations of circulating total cholesterol (TC), low-density lipoprotein (LDL), non-high-density lipoprotein (non-HDL), and triglycerides (TG), and reduced HDL, in addition to other well known risk factors such as obesity, smoking, diabetes, and hypertension, are associated with the development of atherosclerotic disease [2–5]. Elevated lipid concentrations during childhood track into adulthood and increase the risk of CVD, and thus morbidity and mortality [6–9]. Similarly, obesity tracks into adulthood [10, 11], and is positively associated with dyslipidemia [12, 13]. Up to 27% of Danish [13] and 43% of American children and adolescents [14] with overweight/obesity exhibit dyslipidemia, and early treatment and prevention is required [2].

The American Academy of Pediatrics currently recommends that children with obesity or familial aggregation of hyperlipidemia should have their lipid concentrations monitored regularly [15]. Consistent with the increased prevalence of obesity in childhood [16–19], the proportion of children who require screening for dyslipidemia, is increasing.

A prerequisite for the correct identification of dyslipidemia in children and adolescents is the definition of normal ranges of circulating lipid concentrations. Lipid concentrations differ with age, sex, and ethnicity [15], and accordingly age and sex specific reference values from each region/ethnicity is warranted. Current cut-offs for dyslipidemia in children and adolescents are based on data from the North American National Cholesterol Education Program [15]. Based on these cut-offs, 20% of American adolescents exhibit dyslipidemia [20]. A recent European study found similar prevalence of childhood dyslipidemia in a German cohort [21].

The present study aims to identify fasting plasma lipid concentrations from a population-based cohort of Danish/North-European White children and adolescents, and from a normal weight subgroup of this cohort. In addition, the study aims to explore any differences in fasting plasma lipid concentrations between the population-based cohort and children and adolescents with overweight/obesity.

## Methods

### Study populations

From October 2010 until February 2015, a population-based cohort of children and adolescents from schools across 11 municipalities in Denmark were phenotyped (*N* = 2836). This population-based cohort was extended with children 6 − 9 years of age recruited from March 2015 until March 2016 via the public dentistry and health care nurses in one of these municipalities (*N* = 189).

Phenotyping was performed by trained medical staff and involved an extensive questionnaire, a clinical examination, including height and weight, and a fasting blood sample. The questionnaire was completed at home before the examination.

**Fig. 1:**
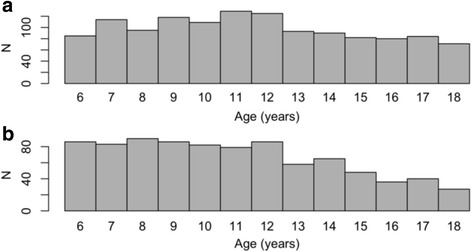
Age distribution. Number of children and adolescents within each age group in the population-based cohort generating the fasting plasma lipid reference intervals. **a** Girls. **b** Boys

Exclusion criteria for this study were 1) no blood samples available (*N* = 112), 2) missing data on lipid concentrations (*N* = 235), 3) more than 30 days between blood sampling and height and weight measurements (*N* = 10), 4) ethnicity other than Danish/North-European White (*N* = 470), 5) known familial hypercholesterolemia (*N* = 0), 6) use of cholesterol-lowering medications (*N* = 0), and 7) age younger than 6 years or older than 19 years (*N* = 57), leaving 2141 participants for further analyses (Fig. 1).

From the population-based cohort, a normal weight subgroup of 1639 (971 girls) children and adolescents was defined by a body mass index (BMI) ≤ 90th and ≥10th percentile for age and sex according to Danish references [22].

From The Children’s Obesity Clinic (TCOC), Department of Pediatrics, Copenhagen University Hospital Holbæk, 1759 children and adolescents with a BMI above the 90th percentile [22] were included in the study from 2008 until December 2015 (the TCOC cohort). These participants had fasting blood samples collected within 30 days of the measurements of height and weight. Exclusion criteria for the present study were 1) ethnicity other than Danish/North-European White (*N* = 248), 2) known genetic causes of obesity (*N* = 16), 3) known familial hypercholesterolemia (*N* = 4), 4) use of lipid-lowering medication (*N* = 0), and 5) age younger than 6 years or older than 19 years (*N* = 70). This left 1421 children and adolescents with overweight/obesity for further analyses.

### Anthropometrics

Height was measured by stadiometer to the nearest 1 mm. Weight was measured to the nearest 100 g on a Tanita® BC418 scale (Tanita Corp., Tokyo, Japan) in the population-based cohort, and on a Tanita® Digital Medical Scale, WB-110 MA (Tanita Corp., Tokyo, Japan) in the TCOC cohort. Measurements were performed wearing light indoor clothes and without shoes. BMI was calculated as weight divided by height in meters squared. The LMS method [23], which uses the median (M), the coefficient of variation (S), and a measure of the skewness (L) in a Box-Cox transformation to normalize the data, was used to calculate the BMI standard deviation scores (SDS) according to Danish references [22].

### Puberty

In the population-based cohort, the pubertal developmental stage according to the Tanner classification [24, 25] was self-evaluated using a descriptive text and color charts for picture pattern recognition. In the TCOC cohort, a pediatrician evaluated the pubertal developmental stage at the first visit to the clinic. Self-evaluated pubertal staging has been shown to adequately describe pre-pubertal versus pubertal status [26]. Therefore, pubertal stage in both the population-based cohort and the TCOC cohort were defined as pre-pubertal (Tanner 1) or pubertal (Tanner 2-5).

### Blood samples

After an overnight fast, venous blood samples were drawn between 7 and 9 AM, processed within 1 h, and analyzed within 6 − 8 h after sampling at the Department of Clinical Biochemistry, Copenhagen University Hospital Holbæk. Analyses were, until May 15th 2013, performed on a Cobas® 6000 (Roche Diagnostics, Mannheim, Germany) (N_population-based_ = 699, N_TCOC_ = 952) and, from May 16th onwards, on a Dimension Vista®1500 (Siemens Healthcare, Erlangen, Germany) (N_population-based_ = 1442, N_TCOC_ = 469) using enzymatic colorimetric method.

Detection limits on the Cobas® 6000 were 0.1 mmol/L for TG and TC, and 0.08 mmol/L for HDL, and on the Dimension Vista®1500, 0.02 mmol/L for TG, 1.29 mmol/L for TC, and 0.05 mmol/L for HDL according to the manufacturer. Internal quality control provided intra-assay coefficients of variations with the following fractions of 0.010 − 0.026 for TC, 0.008 − 0.034 for HDL, and 0.009 − 0.024 for TG on the Cobas® 6000, and of 0.014 − 0.028 for TC, 0.027 − 0.043 for HDL, and 0.010 − 0.020 for TG on the Dimension Vista®1500.

To ensure comparability of TC, HDL, and TG concentrations measured on the Cobas® 6000 and Dimension Vista®1500, routine laboratory data from 2012 and 2014 were extracted from the laboratory information system in order to compare the two analyzers. Only data from adults (age 18 – 99 years) were included (Cobas, 2012: *N* = 37,222/34,744/31,615 and Dimension Vista, 2014: *N* = 33,126/34,436/36,980 for TC/HDL/TG, respectively). Extreme values, defined as over/under the mean +/− 1.5*inter-quartile range, were excluded (Cobas, 2012: *N* = 1404/1749/2343 and Dimension Vista, 2014: *N* = 1262/1801/2600) (Additional file 1: Table S1). The mean of TC concentrations was 0.26 mmol/L and of HDL 0.06 mmol/L lower when measured on the Dimension Vista®1500 compared to the Cobas® 6000. The mean of triglyceride concentrations was 0.08 mmol/L higher on the Dimension Vista®1500 compared to the Cobas® 6000, with a tendency to a slight increase in the difference in higher concentrations (Additional files 2, 3 and 4: Figures S1–S3). To account for these differences in measurement methods, in the present study, TC and HDL concentrations measured with the Cobas® 6000 were deducted by 0.26 and 0.06 mmol/L, respectively. TG concentrations measured on Cobas® 6000 were multiplied by 1.17 and deducted by 0.16 mmol/L. The Friedewald Formula was used to calculate the LDL concentration [27]. Non-HDL was calculated as TC − HDL.

### Dyslipidemia

Dyslipidemia was defined according to the American Heart Associations classification corresponding to the 95th percentile in a American population as total cholesterol >5.2 mmol/L (200 mg/dl), LDL > 3.4 mmol/L (130 mg/dl), HDL <0.9 mmol/L (35 mg/dl), or triglycerides >1.7 mmol/L (150 mg/dl), or a combination thereof [3].

### Statistical methods

All statistical calculations were performed using the statistical software R (version 3.2.4) [28]. The Wilcoxon signed rank test was used to determine differences between sexes in the descriptive statistics. Thereafter, all analyses were performed stratified by sex. Percentiles and smoothed percentile curves were generated using the Generalized Additive Models for Location Scale and Shape (gamlss) package [29] with the penalized cubic spline function and the Box-Cox *t-*distribution family or the Box-Cox Power Exponential distribution family (best fit determined by the Akaike Information Criterion). The models were tested using qq-plots and worm-plots. The effects of puberty were investigated for each sex using generalized linear models adjusted for BMI SDS. In the linear regressions, concentrations of TG were logarithmically transformed to achieve a normal distribution of residuals. Effects of age within each sex were investigated using analysis of variance and Tukey Honest Significant Difference method on normally distributed data, and with the Kruskal-Wallis test and Pairwise Wilcoxon test on non-normally distributed data, with participants grouped in tertiles according to ages in the population-based cohort: young (corresponding to age < 10.1 years in girls. And to age < 9.3 years in boys), middle (corresponding to age > =10.1 and <13.8 years in girls. And to age > =9.3 and <12.7 years in boys), and old (corresponding to age > =13.8 years in girls. And to age > 12.7 years in boys). Normality of data was evaluated using histograms and qq-plots. Differences between mean fasting lipid concentrations between cohorts within age groups for each sex were investigated using Student’s t-test on normally distributed data, and Wilcoxon rank sum test on non-normally distributed data. Odds ratios (OR) were calculated using logistic regression adjusted for age, sex, and pubertal developmental stage.

## Results

From the population-based cohort, 2141 (1275 girls) children and adolescents with a median age of 11.5 years were included in the study. Of these, 17.3% (*N* = 371) were overweight/obese (BMI > 90th percentile) and 6.0% (*N* = 128) were underweight (BMI < 10th percentile) [22]. As there were significant differences between sexes in age, and in concentrations of TC, LDL, non-HDL, and triglycerides (*p* < 0.05), all analyses were performed stratified by sex. Descriptive data on the population-based cohort and the TCOC cohort are presented in Table 1 and percentiles for fasting plasma concentrations of TC, LDL, HDL, non-HDL, and TG﻿ in the population-based cohort in Table 2.

**Table 1:** Descriptive information on the population-based cohort, and The Children’s Obesity Clinic (TCOC) cohort with overweight/obesity

	Population-based		TCOC	
	Girls	Boys	Girls	Boys
N	1275	866	774	647
Age, years	11.9 (6.0 − 19.0)	11.0* (6.1 − 18.9)	11.7 (2.0 − 24.7)	11.8* (2.8 − 21.4)
BMI SDS	0.25** (−3.07 − 3.71)	0.26** (−3.48 − 4.54)	2.7** (1.4 − 6.2)	3.1** (1.38 − 6.1)
TC mmol/L	3.9* (2.0 − 7.5)	3.7** (1.9 − 6.0)	3.9* (1.7 − 6.9)	4.0** (1.7 − 7.2)
LDL mmol/L	2.0** (0.3 − 5.7)	1.9** (0.6 − 4.1)	2.3** (0.5 − 5.8)	2.3** (0.2 − 5.2)
HDL mmol/L	1.5** (0.6 − 2.8)	1.5** (0.5 − 2.7)	1.1** (0.5 − 2.3)	1.1** (0.4 − 2.3)
Non-HDL mmol/L	2.4** (0.4 − 6.1)	2.3** (0.8 − 4.8)	2.8** (1.0 − 6.4)	2.8** (0.6 − 6.0)
TG mmol/L	0.7** (0.1 − 2.8)	0.5** (0.1 − 2.9)	0.9** (0.1 − 4.9)	0.9** (0.1 − 4.6)

Data are medians and ranges. Lipids are fasting plasma concentrations of total cholesterol (*TC*), low-density lipoprotein cholesterol (*LDL*), high-density lipoprotein cholesterol (*HDL*), non-HDL, and triglycerides (*TG*). Significant *P*-values reflect differences between the population-based cohort and the TCOC cohort for each sex. *P < 0.05. **P < 0.0001

**Table 2:** Percentiles for fasting plasma concentrations of TC, LDL, HDL, non-HDL, and TG in the population-based cohort

Age (years)		Girls														Boys													
		6	7	8	9	10	11	12	13	14	15	16	17	18	19	6	7	8	9	10	11	12	13	14	15	16	17	18	19
TC (mmol/L)	2.5th	2.9	2.9	2.9	2.9	2.8	2.8	2.8	2.7	2.7	2.7	2.7	2.7	2.7	2.8	2.6	2.7	2.7	2.7	2.8	2.8	2.7	2.6	2.5	2.4	2.4	2.4	2.4	2.5
	5th	3.1	3.1	3.0	3.0	3.0	3.0	2.9	2.9	2.8	2.8	2.9	2.9	2.9	2.9	2.8	2.8	2.9	2.9	2.9	2.9	2.9	2.8	2.6	2.6	2.5	2.5	2.6	2.6
	10th	3.3	3.3	3.2	3.2	3.2	3.2	3.1	3.1	3.0	3.0	3.0	3.1	3.1	3.1	3.0	3.0	3.1	3.1	3.1	3.1	3.1	3.0	2.8	2.8	2.7	2.7	2.7	2.8
	50th	4.0	4.0	4.0	4.0	4.0	3.9	3.9	3.8	3.8	3.7	3.8	3.8	3.8	3.9	3.7	3.8	3.8	3.9	3.9	3.9	3.9	3.7	3.5	3.4	3.4	3.4	3.4	3.5
	90th	5.0	5.0	5.0	4.9	4.9	4.8	4.8	4.7	4.6	4.6	4.7	4.7	4.7	4.8	4.6	4.6	4.7	4.8	4.8	4.8	4.8	4.6	4.3	4.2	4.2	4.2	4.2	4.3
	95th	5.3	5.3	5.3	5.3	5.2	5.2	5.1	5.0	4.9	4.9	5.0	5.0	5.1	5.1	4.9	4.9	5.0	5.1	5.1	5.1	5.1	4.9	4.6	4.5	4.5	4.4	4.5	4.6
	97.5th	5.6	5.6	5.6	5.6	5.5	5.5	5.4	5.3	5.2	5.2	5.3	5.3	5.4	5.4	5.2	5.2	5.3	5.3	5.4	5.4	5.4	5.1	4.9	4.8	4.7	4.7	4.7	4.8
LDL (mmol/L)	2.5th	1.2	1.2	1.2	1.1	1.1	1.1	1.1	1.0	1.0	1.0	1.0	1.1	1.1	1.1	1.0	1.0	1.0	1.0	1.0	1.0	1.0	1.0	0.9	0.9	0.9	0.9	0.9	1.0
	5th	1.3	1.3	1.3	1.3	1.3	1.2	1.2	1.2	1.2	1.2	1.2	1.2	1.2	1.2	1.2	1.2	1.2	1.2	1.2	1.2	1.2	1.1	1.1	1.1	1.0	1.0	1.1	1.1
	10th	1.5	1.5	1.5	1.5	1.5	1.4	1.4	1.4	1.3	1.3	1.4	1.4	1.4	1.4	1.3	1.3	1.4	1.4	1.4	1.4	1.3	1.3	1.2	1.2	1.2	1.2	1.2	1.2
	50th	2.2	2.2	2.2	2.2	2.1	2.1	2.0	2.0	1.9	1.9	2.0	2.0	2.0	2.1	2.0	2.0	2.0	2.0	2.0	2.0	2.0	1.9	1.8	1.8	1.8	1.8	1.8	1.8
	90th	3.1	3.1	3.1	3.0	3.0	2.9	2.8	2.7	2.7	2.7	2.7	2.8	2.8	2.9	2.7	2.8	2.8	2.8	2.8	2.8	2.8	2.7	2.6	2.5	2.5	2.5	2.5	2.6
	95th	3.4	3.4	3.4	3.3	3.3	3.2	3.1	3.0	3.0	3.0	3.0	3.1	3.1	3.2	3.0	3.0	3.1	3.1	3.1	3.1	3.0	2.9	2.8	2.7	2.7	2.7	2.8	2.8
	97.5th	3.8	3.7	3.7	3.7	3.6	3.5	3.4	3.3	3.3	3.3	3.3	3.4	3.4	3.5	3.3	3.3	3.3	3.4	3.4	3.4	3.3	3.2	3.0	3.0	3.0	3.0	3.0	3.1
HDL (mmol/L)	2.5th	1.0	1.0	1.0	0.9	0.9	0.9	0.9	0.9	0.9	0.9	0.9	0.9	0.9	0.9	1.0	1.0	1.0	1.0	1.0	1.0	1.0	0.9	0.9	0.8	0.8	0.8	0.8	0.8
	5th	1.1	1.1	1.0	1.0	1.0	1.0	1.0	1.0	1.0	1.0	1.0	1.0	1.0	1.0	1.0	1.1	1.1	1.1	1.1	1.1	1.0	1.0	0.9	0.9	0.9	0.8	0.8	0.9
	10th	1.2	1.2	1.1	1.1	1.1	1.1	1.1	1.1	1.1	1.1	1.1	1.1	1.1	1.1	1.1	1.2	1.2	1.2	1.2	1.2	1.1	1.1	1.0	1.0	0.9	0.9	0.9	0.9
	50th	1.5	1.5	1.5	1.5	1.5	1.5	1.5	1.5	1.5	1.4	1.4	1.4	1.4	1.4	1.5	1.5	1.6	1.6	1.6	1.6	1.5	1.5	1.4	1.3	1.3	1.2	1.2	1.2
	90th	2.0	2.0	2.0	2.0	1.9	1.9	1.9	1.9	1.9	1.9	1.9	1.8	1.8	1.8	2.0	2.0	2.0	2.1	2.1	2.0	2.0	1.9	1.8	1.7	1.6	1.6	1.6	1.6
	95th	2.2	2.1	2.1	2.1	2.1	2.1	2.1	2.0	2.0	2.0	2.0	2.0	2.0	2.0	2.1	2.2	2.2	2.2	2.2	2.2	2.2	2.1	1.9	1.8	1.8	1.7	1.7	1.8
	97.5th	2.3	2.3	2.3	2.3	2.2	2.2	2.2	2.2	2.2	2.2	2.1	2.1	2.1	2.1	2.3	2.3	2.3	2.4	2.4	2.4	2.3	2.2	2.1	2.0	1.9	1.9	1.9	1.9
Non-HDL (mmol/L)	2.5th	1.4	1.4	1.4	1.4	1.4	1.4	1.3	1.3	1.3	1.3	1.3	1.4	1.4	1.4	1.2	1.3	1.3	1.3	1.3	1.3	1.3	1.3	1.3	1.2	1.2	1.2	1.2	1.3
	5th	1.6	1.6	1.6	1.6	1.6	1.5	1.5	1.5	1.5	1.5	1.5	1.5	1.6	1.6	1.4	1.4	1.4	1.5	1.5	1.5	1.5	1.4	1.4	1.4	1.4	1.4	1.4	1.4
	10th	1.8	1.8	1.8	1.8	1.8	1.7	1.7	1.7	1.7	1.7	1.7	1.7	1.8	1.8	1.6	1.6	1.6	1.6	1.7	1.7	1.6	1.6	1.6	1.6	1.5	1.5	1.6	1.6
	50th	2.5	2.5	2.5	2.5	2.5	2.5	2.4	2.4	2.4	2.4	2.4	2.4	2.5	2.5	2.2	2.3	2.3	2.3	2.4	2.4	2.3	2.3	2.2	2.2	2.2	2.2	2.2	2.2
	90th	3.4	3.4	3.4	3.4	3.4	3.3	3.3	3.2	3.2	3.2	3.3	3.3	3.4	3.4	3.0	3.1	3.1	3.2	3.2	3.2	3.2	3.1	3.1	3.0	3.0	3.0	3.0	3.0
	95th	3.8	3.8	3.8	3.7	3.7	3.7	3.6	3.5	3.5	3.5	3.6	3.6	3.7	3.8	3.3	3.3	3.4	3.4	3.5	3.5	3.5	3.4	3.3	3.3	3.3	3.2	3.3	3.3
	97.5th	4.1	4.1	4.1	4.1	4.0	4.0	3.9	3.9	3.8	3.8	3.9	4.0	4.0	4.1	3.5	3.6	3.6	3.7	3.7	3.7	3.7	3.6	3.6	3.5	3.5	3.5	3.5	3.5
TG (mmol/L)	2.5th	0.2	0.2	0.3	0.3	0.3	0.3	0.3	0.3	0.3	0.3	0.3	0.3	0.3	0.3	0.2	0.2	0.2	0.2	0.2	0.2	0.3	0.3	0.3	0.3	0.3	0.3	0.3	0.3
	5th	0.3	0.3	0.3	0.3	0.3	0.3	0.3	0.3	0.3	0.4	0.4	0.4	0.4	0.4	0.2	0.2	0.2	0.3	0.3	0.3	0.3	0.3	0.3	0.3	0.3	0.3	0.3	0.4
	10th	0.3	0.3	0.3	0.4	0.4	0.4	0.4	0.4	0.4	0.4	0.4	0.4	0.4	0.5	0.3	0.3	0.3	0.3	0.3	0.3	0.3	0.4	0.4	0.4	0.4	0.4	0.4	0.4
	50th	0.5	0.6	0.6	0.6	0.6	0.6	0.6	0.7	0.7	0.7	0.7	0.7	0.8	0.8	0.5	0.5	0.5	0.5	0.5	0.5	0.6	0.6	0.6	0.6	0.7	0.7	0.7	0.7
	90th	0.9	1.0	1.0	1.0	1.0	1.1	1.1	1.1	1.2	1.2	1.2	1.3	1.3	1.3	0.8	0.8	0.8	0.8	0.9	0.9	1.0	1.0	1.1	1.1	1.1	1.1	1.2	1.2
	95th	1.1	1.1	1.2	1.2	1.2	1.3	1.3	1.3	1.4	1.4	1.5	1.5	1.5	1.6	0.9	1.0	1.0	1.0	1.0	1.1	1.1	1.2	1.3	1.3	1.3	1.4	1.4	1.4
	97.5th	1.3	1.3	1.4	1.4	1.4	1.5	1.5	1.6	1.6	1.7	1.7	1.7	1.8	1.8	1.1	1.1	1.1	1.2	1.2	1.3	1.4	1.4	1.5	1.5	1.6	1.6	1.6	1.7

The 2.5th, 5th, 10th, 50th, 90th, 95th, and 97.5th percentile for each age group in girls and boys are presented

**Fig. 2:**
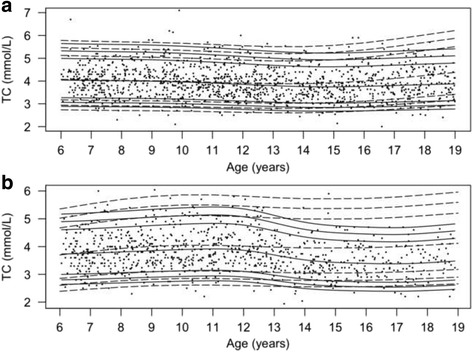
Percentile curves for concentrations of fasting plasma total cholesterol. Smoothed 2.5th, 5th, 50th, 90th, 95th and 97.5th percentile curves for TC in girls (**a**) and boys (**b**). *Full lines* represent the total reference population, and *dotted lines* represent the cohort from The Children’s Obesity Clinic with overweight/obesity. The *dots* represent the participants from the population-based cohort. Concentrations of TC are in mmol/L

**Fig. 3:**
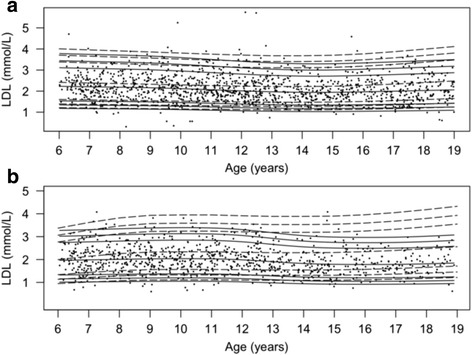
Percentile curves for concentrations of fasting plasma low-density lipoprotein. Smoothed 2.5th, 5th, 50th, 90th, 95th and 97.5th percentile curves for LDL in girls (**a**) and boys (**b**). *Full lines* represent the total reference population, and *dotted lines* represent the cohort from The Children’s Obesity Clinic with overweight/obesity. The *dots* represent the participants from the population-based cohort. Concentrations of LDL are in mmol/L

**Fig. 4:**
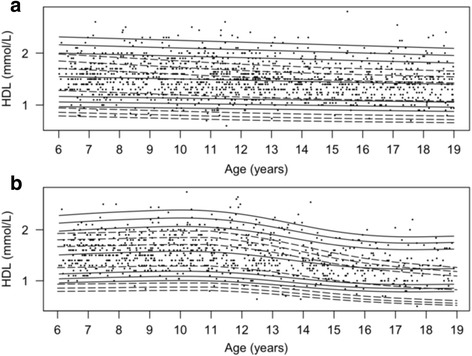
Percentile curves for concentrations of fasting plasma high-density lipoprotein. Smoothed 2.5th, 5th, 50th, 90th, 95th and 97.5th percentile curves for HDL in girls (**a**) and boys (**b**). *Full lines* represent the total reference population, and *dotted lines* represent the cohort from The Children’s Obesity Clinic with overweight/obesity. The *dots* represent the participants from the population-based cohort. Concentrations of HDL are in mmol/L

**Fig. 5:**
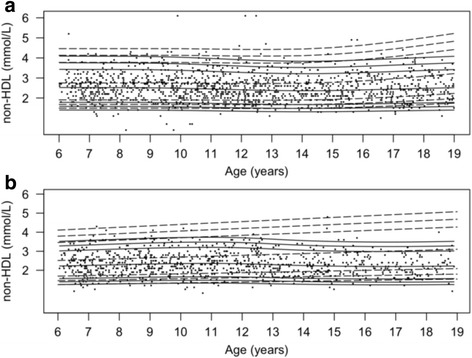
Percentile curves for concentrations of fasting plasma non-high-density lipoprotein. Smoothed 2.5th, 5th, 50th, 90th, 95th and 97.5th percentile curves for non-HDL in girls (**a**) and boys (**b**). *Full lines* represent the total reference population, and *dotted lines* represent the cohort from The Children’s Obesity Clinic with overweight/obesity. The *dots* represent the participants from the population-based cohort. Concentrations of non-HDL are in mmol/L

**Fig. 6:**
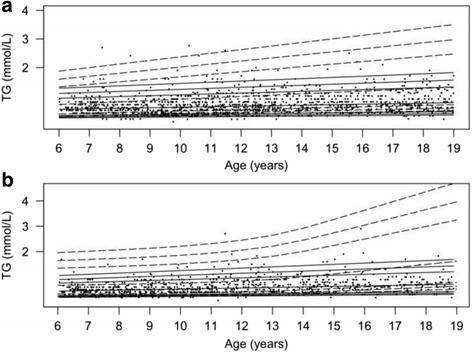
Percentile curves for concentrations of fasting plasma triglycerides. Smoothed 2.5th, 5th, 50th, 90th, 95th and 97.5th percentile curves for TG in girls (**a**) and boys (**b**). *Full lines* represent the total reference population, and *dotted lines* represent the cohort from The Children’s Obesity Clinic with overweight/obesity. The *dots* represent the participants from the population-based cohort. Concentrations of TG are in mmol/L

The percentile curves along with the data-points from the population-based cohort are provided in Figs. 2, 3, 4, 5 and 6. In addition, percentile curves for the TCOC cohort are shown in the corresponding graphs (Figs. 2, 3, 4, 5 and 6). The same percentiles for the normal weight subgroup of the population-based cohort are presented in Additional file 5: Table S2. The corresponding centiles in the total population for the most part differed from the normal weight subgroup on the second decimal of the lipid concentrations; there was a tendency for a difference on the first decimal of 0.1 in the upper centiles.

### Fasting plasma total cholesterol

In girls, the TC concentrations (Fig. 2a) were 0.1 mmol/L higher in the youngest tertile compared to the middle tertile (95% confidence interval (CI): [0.01 − 0.22], *P* = 0.03) and 0.2 mmol/L higher compared to the oldest tertile (95% CI: [0.23 − 0.33], *P* = 2.3*10^−6^). Mean concentrations in girls in the TCOC cohort differed from the population-based cohort with higher values only in the oldest age group (*P* = 0.0018). In boys, the TC concentration (Fig. 2b) was 0.4 mmol/L lower in the oldest tertile compared to the youngest tertile (95% CI: [0.23 − 0.48], *P <* 0.00001) and 0.5 mmol/L lower compared to the middle tertile (95% CI: [0.34 − 0.59], *P <* 0.00001). The levels of TC in boys from the TCOC cohort were stable and higher than in boys in the population-based cohort in the middle and oldest age groups (*P* < 0.03).

### Fasting plasma LDL

In girls, the LDL concentrations (Fig. 3a) were 0.1 mmol/L higher in the youngest population-based tertile compared to the middle tertile (95% CI: [0.04 − 0.24], *P* = 0.0032) and 0.2 mmol/L higher compared to the oldest tertile (95% CI: [0.11 − 0.30], *P* = 2.7*10^−6^), but remained stable at a higher level throughout all ages in the TCOC cohort (*P <* 0.002). The concentrations of LDL (Fig. 3b) in boys were 0.2 mmol/L higher in the youngest tertile compared to the oldest tertile (95% CI: [0.10 − 0.32, *P* = 1.8*10^−5^), and 0.3 mmol/L higher (95% CI:[0.15;0.37], *P* < 0.000001) compared to the middle tertile. The LDL concentrations in boys from the TCOC cohort were higher than in boys in the population-based cohort at all ages (*P <* 0.0001).

### Fasting plasma HDL

In girls, the HDL concentrations (Fig. 4a) in the population-based cohort were 0.1 mmol/L lower in the oldest tertile compared to the middle tertile (95% CI: [0.02 − 0.12], *P* = 0.004) and 0.1 mmol/L lower compared to the youngest tertile (95% CI: [0.04 − 0.15], *P* = 4.4*10^−5^). In girls from the TCOC cohort, the HDL concentrations exhibited a constantly lower level (*P <* 2.2*10^−16^) in all three age groups. The HDL concentrations (Fig. 4b) in boys in the population-based cohort in the oldest tertile were 0.2 mmol/L lower compared to the youngest tertile (95% CI: [0.16 − 0.29], *P <* 0.00001) and 0.2 mmol/L lower compared to the middle tertile (95% CI:[0.18;0.31], *P* > 0.00001). In boys, the HDL concentrations in the TCOC cohort were lower than in the population-based cohort at all ages (*P <* 6.0*10^−15^) and were significantly higher in the youngest age group compared to the oldest age group (95% CI: [0.17 − 0.30], *P* = 3.6*10^−12^).

### Fasting plasma non-HDL

In girls, the concentration of non-HDL (Fig. 5a) did not differ between the three age groups in the population-based cohort (*P* > 0.05). In girls from the TCOC cohort, the non-HDL concentrations exhibited a constantly higher level in all three age groups compared to the population-based cohort (*P* < 0.0001). In boys, the concentration of non-HDL was 0.2 mmol/L lower in the oldest tertile compared to the middle tertile (95% CI:[0.09;0.32], *P* = 0.00012). No difference was observed between the youngest tertile and the middle or oldest tertile (*P* > 0.05). Concentrations of non-HDL in boys in the TCOC cohort were higher in all age groups (*P* < 2.4*10^−6^).

### Fasting plasma triglyceride

TG concentrations increased from the youngest to the middle tertile in girls (*P* = 2.4*10^−5^) and boys (*P* = 0.00042), and from the middle to the oldest tertile in girls (*P* = 0.0011) and in boys (*P* = 1.8*10^−5^) (Fig. 6a and b). TG concentrations in both girls and boys from the TCOC cohort were higher than in the population-based cohort at all ages (*P <* 2.2*10^−14^). Furthermore, in girls from the TCOC cohort, concentrations of TG were higher in the oldest age group compared to the middle (*P* = 2.0*10^−5^) and the youngest (*P* = 1.4*10^−12^) age groups, and, in boys from the TCOC cohort, higher in the oldest age group compared to the middle (*P =* 0.035) and youngest age group (*P* = 1.1*10^−9^).

### Puberty

Data on pubertal developmental stages were available in 77% (*N* = 984) of girls and in 53% (*N* = 463) of boys in the population-based cohort. Pubertal girls exhibited a 0.2 mmol/L lower TC concentration compared to pre-pubertal girls (95% CI:[−0.25; −0.06], *P* = 0.0013). Pubertal girls exhibited a 0.1 mmol/L lower LDL concentration compared to pre-pubertal girls (95% CI:[−0.21; −0.04], *P* = 0.0030). Pubertal girls exhibited a 0.1 mmol/L lower concentration of HDL compared to pre-pubertal girls (95% CI:[−0.11; −0.02], *P* = 0.0028). No difference was observed in the concentrations of non-HDL in pubertal girls compared to pre-pubertal girls (95% CI:[−0.14;0.04], *P* = 0.25). The concentration of TG was 15% higher in pubertal girls compared to pre-pubertal girls (95% CI:[1.08;1.22], *P* = 6.2*10^−6^).

In boys, puberty was associated with a 0.2 mmol/L lower concentration of TC (95% CI:[−0.37; −0.11], *P* = 4.2*10^-4^), a 0.2 mmol/L lower concentration of HDL (95% CI:[−0.24; −0.11], *P* = 2.3*10^-7^), and a 12% higher TG concentration (95% CI:[1.03;1.22], *P* = 0.0078) compared to pre-puberty. No differences were observed in the concentrations of LDL (95% CI:[−0.21;0.01], *P* = 0.071) or non-HDL (95% CI:[−0.17;0.06], *P* = 0.34) between pubertal and pre-pubertal boys. Adjusting for age in the generalized linear models rendered all associations between lipid concentrations and puberty in girls and in boys insignificant.

Similar effects of puberty were observed in the TCOC cohort among girls as well as boys.

### Dyslipidemia

The prevalence of dyslipidemia was 6.4% in the population-based cohort, and 28.0% in the TCOC cohort (Table 3). The odds ratio (OR) for exhibiting dyslipidemia was 6.2 (95% CI: 4.9 − 8.1, *P* < 2*10^−16^) in the TCOC cohort compared to the population-based cohort. In the population-based group, the OR for a child or adolescent with a BMI > 90th percentile to exhibit dyslipidemia was 2.8 (95% CI: 1.8 − 4.4, *P* = 4.9*10^−6^) compared to a child with a BMI <90th percentile, when adjusting for age, sex, and pubertal developmental stage. The OR for a child or adolescent from the TCOC cohort compared to a child or adolescent from the population-based cohort with a BMI > 90th percentile to have dyslipidemia was 2.9 (95% CI: 2.0 − 4.4, *P* = 1.0*10^−7^). All ORs were driven by differences in BMI SDS, and became insignificant when further adjusting for BMI SDS in the models (*P* > 0.05).

**Table 3:** Prevalence of dyslipidemia

	TCOC N (%)	Population-based cohort N (%)	Subgroup of normal weight individuals N (%)
TC > 5.2 mmol/L	101 (7.1%)	71 (3.3%)	46 (2.8%)
LDL > 3.4 mmol/L	97 (6.8%)	49 (2.3%)	33 (2.0%)
HDL < 0.9 mmol/L	180 (12.7%)	35 (1.6%)	21 (1.3%)
TG > 1.7 mmol/L	211 (14.8%)	27 (1.3%)	14 (0.8%)
One or more of above	398 (28.0%)	137 (6.4%)	86 (5.2%)

## Discussion

This study provides reference values for fasting plasma concentrations of TC, LDL, HDL, non-HDL, and TG in a population-based cohort of Danish/North-European White children and adolescents. In addition, the study evaluates the association of overweight and obesity with these variables. We calculated the percentiles in both the total population-based cohort and in a normal-weight subgroup of the cohort. Excluding the individuals with overweight/obesity in the population-based cohort did not change the reference curves markedly other than on the first decimal in some of the upper percentiles. This offers no clinical relevance, since the accuracies of the analysis methods exhibit a higher variation than the differences in values between the total population-based cohort and the normal-weight subgroup. We therefore generated reference percentiles for the entire population-based cohort.

Except for the concentrations of TG, the lipid concentrations varied more throughout childhood and adolescence in boys compared to girls, with the reference curves in boys exhibiting a biphasic pattern for TC, LDL, HDL, and non-HDL, and a steady increase for TG. The reference levels at the 50th and 90th percentile in both a French cohort (*N* = 1976 (1004 girls), age 7 − 20 years) and a German cohort (*N* = 2571 (1226 girls), age 0 − 16 years), albeit showing comparable patterns throughout childhood, exhibited higher concentrations for TC, LDL, and TG, but similar levels of the HDL concentrations in both sexes compared to our study [21, 30]. Differences in overweight/obesity prevalences do not explain this difference, as the prevalences of overweight and obesity in Germany, France, and Denmark are comparable [18, 31, 32]. In addition, only lean subjects were included in the French study [30], and in the present study, the 50th percentile did not change, when including the underweight, overweight, and obese individuals from the population-based cohort.

Non-HDL concentrations among children and adolescents are more indicative of persistent dyslipidemia compared to TC, LDL, or HDL [5], and in a meta-analysis of 233,455 adults a more potent marker of cardiovascular risk than LDL [33]. In our study, concentrations of non-HDL were not affected by puberty in either sex, and in girls not affected by age. In boys, concentrations were lower in the oldest age group of boys compared with the middle age group of boys. In contrast, a study in Slovakian children and adolescents found a negative effect of age on non-HDL in both girls and boys [34].

Through all ages and in both sexes in the present study, children from the TCOC cohort exhibited higher concentrations of TC, LDL, non-HDL, and TG, and lower concentrations of HDL than the population-based cohort, underlining the reports from a previous study on the effects of obesity on fasting plasma lipid concentrations [13, 35]. In the German cohort, the prevalence of dyslipidemia was similar to what is previously described in US children and adolescents (*N* = 1482 (725 girls), age 8 − 17 years) [20, 21]. The prevalence of dyslipidemia in our cohort was considerably lower than described in both of these cohorts. The difference to the German cohort may in part be explained by their inclusion of a cohort with obesity from an obesity clinic, where the degree of obesity may have been higher than the overall degree of obesity in the population, even though the prevalence of obesity in their cohort resembled the overall German prevalence of obesity [21]. In the present study, a higher OR for dyslipidemia was observed in the TCOC cohort compared to the subgroup with overweight/obesity from the population-based cohort; a difference that was correlated with differences in BMI SDS. This indicates that differences in the degree of obesity may influence the prevalence of dyslipidemia in a given cohort. Similarly, the difference in prevalences of dyslipidemia between our cohort and the American cohort may be explained by the difference in the prevalences of obesity, being higher in USA and with increasing prevalence of extreme obesity [17–19]. In the present study we focused on fasting concentrations of lipids and dyslipidemia. Future studies should investigate the distribution and effect of obesity on other cardiovascular risk factors such as hypertension and insulin resistance, and the possible effects – individually and combined – of multiple risk factors present concomitantly in children and adolescents.

A limitation to our study was the lack of diet registration. Nevertheless, adherence to the fasting state was assured by interview on the morning of the blood sampling. If the participant was not fasting, another appointment for blood sampling was made.

During the study period, methods for blood sample analysis changed at the laboratory that analyzed our samples. This was due to hospital administrative decisions not related to this study. The comparability of the results was ensured, by applying a quality control analysis on all measurements performed at the laboratory in 2012 (Cobas® 6000) and 2014 (Dimension Vista®1500). The variation between the Cobas® 6000 and the Dimension Vista®1500 in measured concentrations of HDL and TG was less than the intra-assay variation, indicating that it may not have been necessary to apply a correction factor on these variables. Accordingly, applying the conversion factors on the lipid concentrations did not change any of the conclusions in the study.

In the population-based sample, the pubertal developmental stage was self-evaluated with picture pattern recognition due to practical considerations. Thus, this method of determining puberty might be less accurate than the same evaluation done by a trained pediatrician; however, self-evaluated puberty distinguishing between the pre-pubertal and pubertal stage has been shown to be reliable in epidemiological studies [26]. The effects of puberty on lipid concentrations did not differ from the effects of age in our cohorts. Thus, we consider age to be the most straightforward way to define references for fasting plasma lipid concentrations in Danish/North-European children and adolescents. The references defined by the present study are presented both as charts easy to use for the clinicians plotting the values of their patients and monitoring progressions, and in tables for integration into electronic laboratory systems.

The primary strength of the study was a large cohort of healthy Danish children recruited from schools, public dentists, and healthcare nurses in 11 municipalities in Denmark, with an extensive phenotype collected alongside biochemical markers. In addition, the TCOC cohort, a cohort of children and adolescents with overweight/obesity, was collected during the same period of time and provided information on the effects of obesity on the lipid concentrations, elucidating the health-related impact obesity has on human metabolism at an early age.

## Conclusion

This study provides reference values for fasting plasma lipid concentrations for both sexes in the age range 6 − 19 years in Danish/North-European whites. Different patterns for girls and boys were observed for lipid concentrations throughout childhood and adolescence. Furthermore, the study evaluates the effects of obesity on lipid concentrations in childhood including an increased risk of dyslipidemia, which might predispose the children with obesity to premature CVD morbidity and mortality later in life.

## Additional files

Additional file 1: Table S1. Summary of routine laboratory data on fasting plasma concentrations of TC, HDL, and TG from 2012 and 2014. (XLSX 8 kb)

Additional file 2: Figure S1. Cumulative relative frequency plots of concentrations of TC. Concentrations measured in 2012 measured on the Cobas® 6000 (Blue curve) and in 2014 on the Dimension Vista®1500 (Red curve). (PDF 29 kb)

Additional file 3: Figure S2. Cumulative relative frequency plots of concentrations of HDL. Concentrations measured in 2012 measured on the Cobas® 6000 (Blue curve) and in 2014 on the Dimension Vista®1500 (Red curve). (PDF 28 kb)

Additional file 4: Figure S3. Cumulative relative frequency plots of concentrations of TG. Concentrations measured in 2012 measured on the Cobas® 6000 (Blue curve) and in 2014 on the Dimension Vista®1500 (Red curve). (PDF 31 kb)

Additional file 5: Table S2. The 2.5th, 5th, 10th, 50th, 90th, 95th, and 97.5th percentiles for lipid concentrations in girls (*N* = 971) and boys (*N* = 668) with 10th ≤ BMI SDS ≤ 90th at ages 6–19 years. (XLSX 44 kb)

Additional file 6: Table S3. Routine laboratory data on fasting plasma concentrations of TC, HDL, and TG from 2012. Age in years. Concentrations in mmol/L. (CSV 4181 kb)

Additional file 7: Table S4. Routine laboratory data on fasting plasma concentrations of TC, HDL, and TG from 2012. Age in years. Concentrations in mmol/L. (CSV 4279 kb)

Additional file 8: Table S5. Dataset from the population-based and the TCOC cohort. Age in years. Concentrations in mmol/L. Affiliation 1 and 5: children in the TCOC cohort. Affiliation 3: children in the population-based cohort. Interval: days between blood sampling and anthropometrics. Tanner_girl: Pubertal stage 1–5 according to the Tanner classification. Tanner_boy: Pubertal stage 1–5 according to Tanner classification. (CSV 179 kb)

## Abbreviations

BMI SDS: Body mass index standard deviation score
CVD: Cardiovascular disease
HDL: High-density lipoprotein
LDL: Low-density lipoprotein
TC: Total cholesterol
TG: Triglycerides
