# Supplementary figures and images for: Dyslipidemia and reference values for fasting plasma lipid concentrations in Danish/North-European White children and adolescents
Source: BMC Pediatr. 2017 Apr 28;17:116. doi: 10.1186/s12887-017-0868-y (PMC5410076; doi:10.1186/s12887-017-0868-y)

Total cholesterol

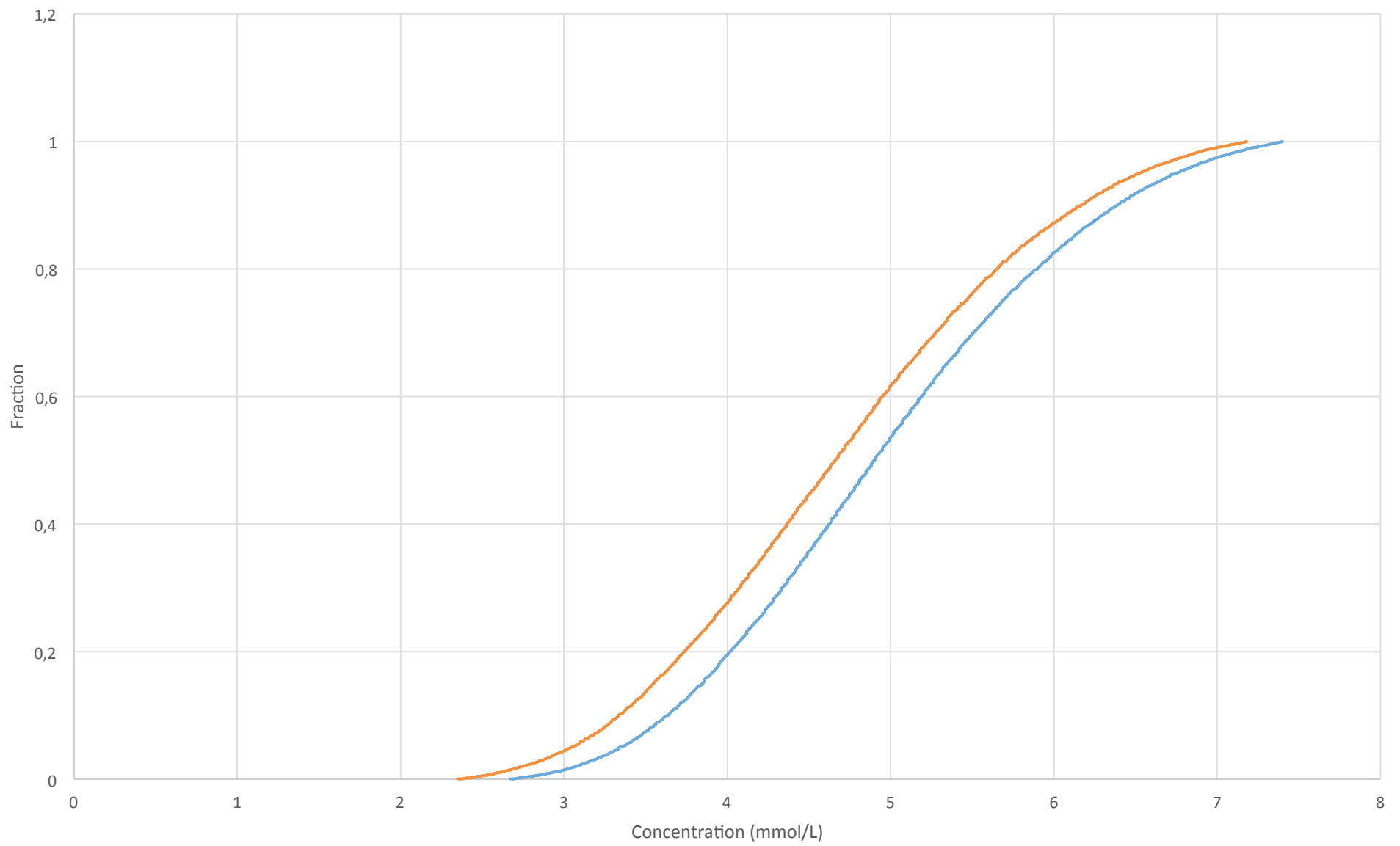

Supplement: Supplementary file 2 — Cumulative relative frequency plots of concentrations of TC. Concentrations measured in 2012 measured on the Cobas® 6000 (Blue curve) and in 2014 on the Dimension Vista®1500 (Red curve). (PDF 29 kb) [file 12887_2017_868_MOESM2_ESM.pdf]

# HDL

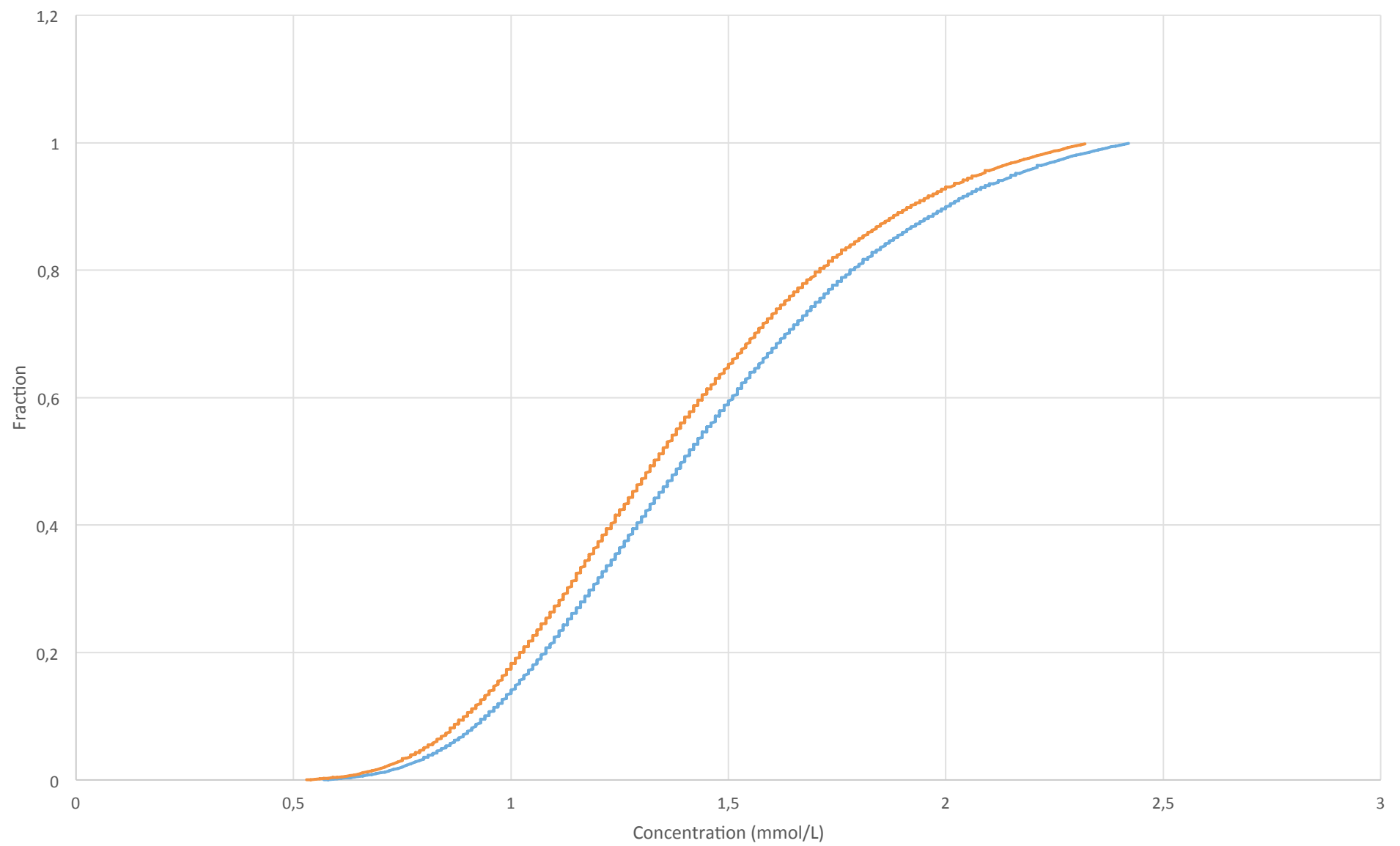

Supplement: Supplementary file 3 — Cumulative relative frequency plots of concentrations of HDL. Concentrations measured in 2012 measured on the Cobas® 6000 (Blue curve) and in 2014 on the Dimension Vista®1500 (Red curve). (PDF 28 kb) [file 12887_2017_868_MOESM3_ESM.pdf]

# Triglycerides

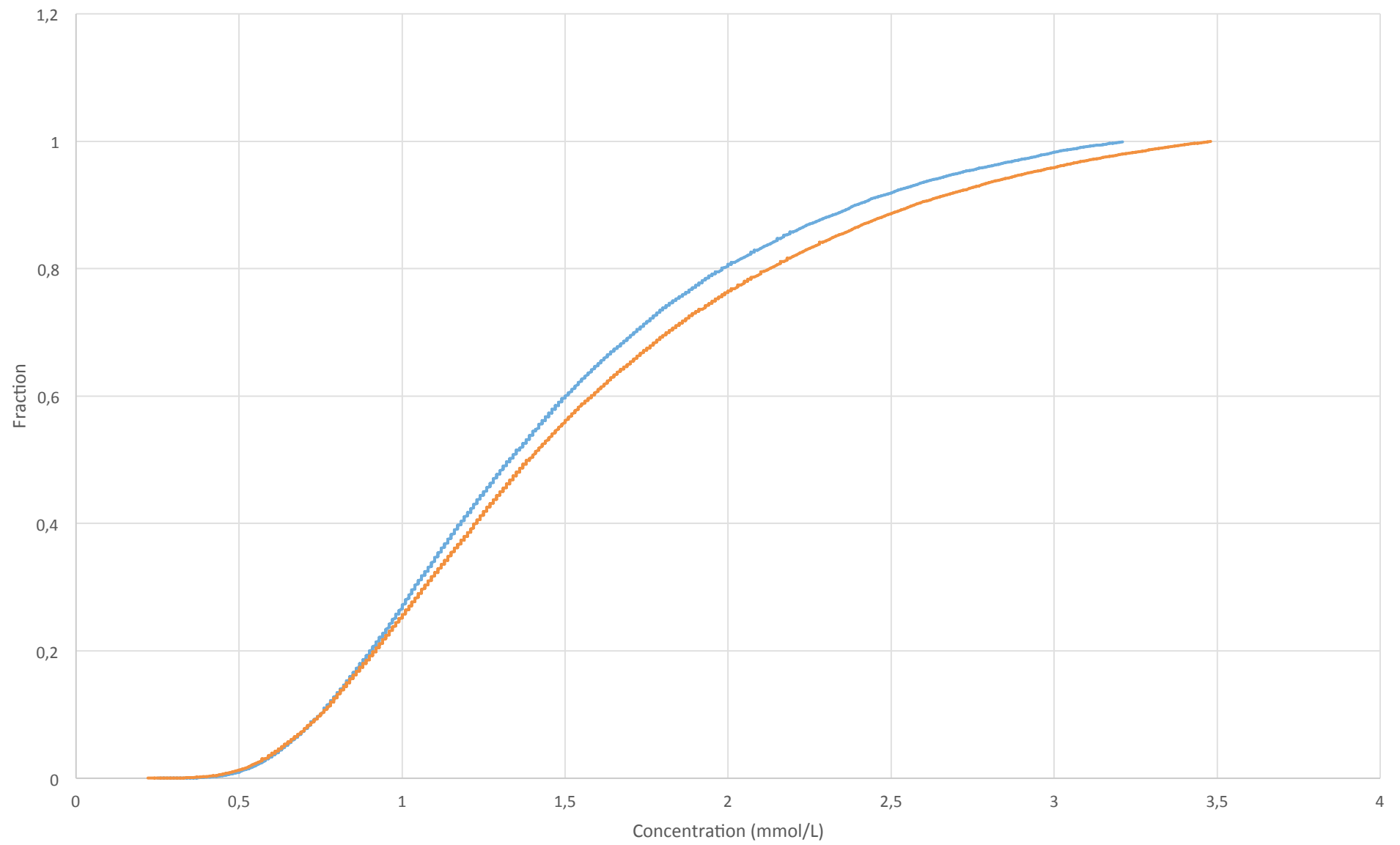

Supplement: Supplementary file 4 — Cumulative relative frequency plots of concentrations of TG. Concentrations measured in 2012 measured on the Cobas® 6000 (Blue curve) and in 2014 on the Dimension Vista®1500 (Red curve). (PDF 31 kb) [file 12887_2017_868_MOESM4_ESM.pdf]
